# Grip Strength as an Indicator of Health-Related Quality of Life in Old Age—A Pilot Study

**DOI:** 10.3390/ijerph14121447

**Published:** 2017-11-24

**Authors:** Christina Musalek, Sylvia Kirchengast

**Affiliations:** Department of Anthropology, University of Vienna, Althanstrasse 14, A-1090 Vienna, Austria; tinamusalek@gmx.at

**Keywords:** grip strength, muscle strength, old age, health related quality of life, intergenerational contacts, social contacts

## Abstract

Over the last century life expectancy has increased dramatically nearly all over the world. This dramatic absolute and relative increase of the old aged people component of the population has influenced not only population structure but also has dramatic implications for the individuals and public health services. The aim of the present pilot study was to examine the impact of physical well-being assessed by hand grip strength and social factors estimated by social contact frequency on health-related quality of life among 22 men and 41 women ranging in age between 60 and 94 years. Physical well-being was estimated by hand grip strength, data concerning subjective wellbeing and health related quality of life were collected by personal interviews based on the WHOQOL-BREF questionnaires. Number of offspring and intergenerational contacts were not related significantly to health-related quality of life, while social contacts with non-relatives and hand grip strength in contrast had a significant positive impact on health related quality of life among old aged men and women. Physical well-being and in particular muscle strength—estimated by grip strength—may increase health-related quality of life and is therefore an important source for well-being during old age. Grip strength may be used as an indicator of health-related quality of life.

## 1. Introduction

Human life expectancy has increased dramatically over the last 100 years. During the last four decades human life expectancy at birth rose more than one-third and this trend is predicted to continue. By 2050 it is expected that nearly 1.5 billion people worldwide will be older than 65 years [1,2,3]. Consequently the absolute and relative amount of older people is increasing rapidly all over the world [4]. This demographic trend is a challenge, not only for communities, economies, governments, social systems, and public health services but also for families and individuals [5]. This is mainly due to the fact that although nothing seems to be worthier than the fact that people can live longer, it is still a challenge to preserve a high health-related quality of life during old age. In many cases health-related quality of life is impaired because old age is associated with adverse somatic changes [6,7,8,9,10] which may increase the risk of diseases and vulnerabilities resulting in a reduced quality of life [11,12]. But what does quality of life mean? During the last decades the evaluation of quality of life among older adults has become increasingly important in health as well as in social sciences. The concept of quality of life was introduced in the seventies of the last century as a key term in medical indexes and in 1991 the WHO started to developed a unifying and transcultural definition of quality of life. They defined it as *“the individual’s perception of his or her position in life, within the cultural context and value system he or she lives in, and in relation to his or her goals, expectations, parameters and social relations. It is a broad ranging concept affected in a complex way by the person’s physical health, psychological state, level of independence, social relationships and their relationship to salient features of their environment”* [13]. Based on this definition the concept of health-related quality of life was introduced, which is a broad and multidimensional construct that includes various domains of physical, psychological and social health. Health-related quality of life is clearly influenced by several endogenous and exogenous parameters [10,14,15,16,17]. An important endogenous parameter of health-related quality of life is physical well-being or physical fitness [18]. Especially the loss of muscle strength which enhances the risk of fatal falls and consequently the risk of fractures is strongly related to reduced health-related quality of life [19,20,21,22]. A useful proxy to determine physical fitness and physical well-being, especially among elderly people, is the measurement of hand grip strength, which is increasingly used as an indicator of overall muscle strength and function [23,24]. A high grip strength is strongly associated with preserved mobility [25], higher activities of daily living [26,27] and decreased disability [28,29]. Beside the effects of physical well-being, health-related quality of life is influenced by environmental and social factors [30]. Well working social networks and social contacts enhance wellbeing and health-related quality of life during old age [6,17,31,32,33]. For a long time social contacts during old age were mainly provided by close relatives, especially offspring. Unfortunately over the past few decades Western societies have undergone dramatic changes not only in the family structure, but also the relationships between older parents and their adult children. On the one hand as life expectancy increases and so the absolute number of old aged people increases in all societies, on the other hand the number of offspring has decreased and many people remain voluntarily or involuntarily childless. This trend adds burdens to families and welfare systems, the two major pillars of support in old age [34,35]. Since the 1970s the quality and quantity of social relationships, especially between close relatives have been increasingly recognized as important factors of high health-related quality of life and subjective well-being during old age [36]. Although several studies have indicated the generally positive effect of social support, and membership in a family on health and survival during old age [36,37,38,39,40,41,42,43], some studies have reported no association between well-being of the elderly and the contact frequency with their offspring [32,44,45,46,47]. In general the preservation of health, physical fitness and physical wellbeing, social contacts, an active life style and consequently a high health-related quality of life during old age should be a central goal of each society confronted with an increasing life expectancy. It is absolutely necessary to identify factors enhancing a positive ageing process and indicators of health related quality of life. In Austria life expectancy had increased dramatically during the 20th century. Therefore the present pilot study focuses on the impact of physical well-being estimated by hand grip strength and social contact frequency (relatives and non-relatives) on health-related quality of life for an Austrian sample for the first time.

## 2. Materials and Methods

### 2.1. Participants

Sixty three subjects ranging in age between 60 and 94 years (mean = 72.5 ± 9.0) were enrolled in the present pilot study. The sample comprised 22 men aged between 60 and 84 years (mean = 72.5 ± 7.4) and 41 women aged between 60 and 94 years (mean = 72.5 ± 10.1). Without any doubt this sample size is extremely small and limits any interpretations of the results. Originally we planned to recruit 100 women and 100 men for this pilot study. The subjects were recruited via a snowball system and broadcasting. Unfortunately less than 100 participants willing to participate in the present study were identified. Especially among the 80 years and older age group recruitment proved extremely difficult. On the one hand the examination was too long for many potential participants, on the other hand it was nearly impossible to recruit participants of the age group meeting all the inclusion criteria listed below. Furthermore several participants aborted the interviews. Since the time schedule for the data collection of this pilot study was restricted to three months, it was not possible to increase the number of participants. Therefore we decided to carry out this pilot study based on a very small sample. The following inclusion criteria have been defined:

1. a stable medical condition;
2. no need of intensive care;
3. independence in performing daily living activities;
4. active life style;
5. sufficient mental capacity and cognitive function to answer the questions;
6. willingness to participate in the study.

All participants originated from Austria.

### 2.2. Procedure

After a first phone contact individual appointments were organized. Data collection took place mainly at the private homes of the participants, at social centres, meeting rooms of senior associations and a home for the elderly. Before data collection started all participants were informed about the objectives and methodology of the study. Beside the objectives of the study, the right to withdraw at any time was explained. Strict confidentiality was assured. The study was conducted in compliance with the Ethical Principles for Medical Research Involving Human Subjects of the Helsinki Declaration. Data collection took place by means of face to face interviews based on structured questionnaires and carried out by a trained interviewer. Additionally hand grip strength was measured for the right and left hand.

### 2.3. Questionnaires

Before starting data collection a pre-testing phase was carried out on fifteen elderly subjects in order to screen for potential problems in the questionnaire. The questionnaire was divided into three parts. In the first part sociodemographic and medical information was gathered. The second part focused on reproductive history and intergenerational and other social contacts. Part three comprised the German language version of WHOQOL-BREF.

Each data collection event started with an interview regarding sociodemographic parameters such as educational level, professional training, marital status, living situation (alone versus in a partnership) and place of residence. Furthermore medical history was documented. In detail current acute and chronic diseases as well as regular medication were documented. Furthermore data concerning previous diseases and surgeries was collected. Reproductive history and intergenerational contact information covered a diverse set of parameters, and the number, age and sex of offspring, including sons and daughters and grandchildren was recorded. Additionally the frequency of intergenerational contacts was documented. Contact frequency was expressed by the number of personal contacts (meetings, social media and phone contacts) per an average month for each offspring. The average contact frequency with each offspring was summarized to the variable contact frequency with relatives per month. Furthermore the contact frequency per month (meetings, social media and phone contacts) with non-relatives (neighbours, friends, like-minded people, but also nurses) was documented. The contact frequency with each non-relative was summarized to the variable contact frequency with non-relatives per month.

### 2.4. WHOQOL-BREF

Health-related quality of life was determined by means of the standardized WHOQOL-BREF. [48]. Based on the WHO definition of health-related quality of life the WHO developed a 100-item quality of life inventory (QOL), the so called WHOQOL-100, in order to assess health-related quality of life in a standardized way [13]. To ensure a worldwide validity the WHOQOL-100 was developed simultaneously in 15 field centres around the world. The important aspects of quality of life and ways of asking about quality of life were drafted based on the statements by patients with a range of diseases, by healthy people and by health professionals in a variety of cultures. The WHOQOL-100 was rigorously tested to assess its validity and reliability in each of the field centres [13]. In the present pilot study health-related quality of life was determined by the short version of the WHOQOL-100, the so called WHOQOL-BREF. The WHOQOL-BREF represents an abbreviated 26 item version of the WHOQOL-100, which provides a valid and reliable alternative to the assessment of domain profiles using the WHOQOL-100. The high reliability and validity of the WHOQOL-BREF was shown for several populations worldwide [49,50,51,52,53]. In detail, the WHOQOL-BREF contains two items from the Overall Quality of Life and General Health facet and one item from each of the remaining 24 facets [13]. These facets are categorized into four main domains: Physical capacity (DOM I) comprising seven items (activities of daily living, dependence on medicinal substances and medical aids, energy and fatigue, mobility, pain and discomfort, sleep and rest, work capacity), Psychological Well-being (DOM II) comprising six items (bodily image and appearance, negative feelings, positive feelings, self-esteem, spirituality/religion/personal beliefs, thinking, learning, memory and concentration), Social Relationships (DOM III) comprising three items (personal relationships, social support, sexual activity) and Environment (DOM IV) comprising eight items (financial resources, freedom, physical safety and security, health and social care: accessibility and quality, home environment, opportunities for acquiring new information and skills, participation in and opportunities for recreation/leisure activities, physical environment (pollution/noise/traffic/climate), transport. Additionally the global domain is determined (subjective quality of life, subjective health status). All items were rated on a 5-point scale with a higher score indicating a higher quality of life. Domain scores were calculated by multiplying the mean of all facet scores included in each domain by a factor of 4 and accordingly, potential scores for each domain ranged from 4 to 20. In the present study the German version of the WHOQOL-BREF according to Angermeyer et al. [48] was used.

### 2.5. Hand Grip Strength

Grip strength has become a popular indicator of physical functioning in surveys [24,25,26,54,55,56]. Measurements were performed with both hands using a hand-hold calibrated dynamometer (JAMAR, Hatfield, PA, USA). The dynamometers were calibrated at the start of the study. Grip strength was measured in a face-to-face assessment with the participant sitting comfortably. The examiner ensured that the arm to be tested was held by their side and their elbow was at a 90° angle. The participant was asked to squeeze the hand as hard as possible for few seconds. The measurement was repeated after a recovery period of 5 min in order to test the reliability. Hand grip strength was expressed in kilograms (kg). For further analyses exclusively the grip strength of the dominant hand was used.

### 2.6. Statistical Analyses

Statistical analyses were carried out using SPSS for Windows Version 22.0 (SPSS Inc., Chicago, IL, USA). Descriptive statistics in particular means, standard deviations, range, absolute and relative frequencies were calculated for better sample description. The total score of health related quality of life was calculated by summarizing the scores of all five domains. The tertiles of this total score were computed and three categories of health-related quality of life defined: lowest tertile = low health-related quality of life. Medium tertile = medium health-related quality of life and highest tertile = highest health-related quality of life. Non-parametric Mann-Whitney tests, Kruskall-Wallis-tests and χ^2^-tests were computed in order to test group differences with respect to their statistical significance. Spearman correlations were computed test to correlations patterns between health-related quality of life, grip strength and contact frequency. Additionally multiple regression analyses were performed to test the impact of hand grip strength as well as sociodemographic parameters and intergenerational and social contact frequency on health-related quality of life.

## 3. Results

### 3.1. Sample Description and Gender Differences

A detailed description of sociodemographic parameters is given in Table 1. Male and female participants differed neither in age, nor in educational level, marital status and living situation significantly. Men surpassed women significantly (*p* < 0.05) in the number of children. No statistically significant differences were found for number of grandchildren and contact frequency (see Table 1). As to be expected male participants surpassed their female counterparts in hand grip strength significantly (*p* < 0.01) Concerning health-related quality of life men showed higher scores in the physical and psychic domain, while women exhibited higher scores of the social as well as the environmental domain. The sex differences in health related quality of life however, were not of statistical significance.

### 3.2. Age Effects on Health Related Quality of Life and Grip Strength

As demonstrated in Table 2 hand grip strength decreases significantly (<0.001) with increasing age in women. Among men hand grip strength decreases with increasing age only insignificantly. Concerning health-related quality of life domains a decrease of all domain scores with increasing age was observed. Among women the scores of the global, physical and psychic domain decreased significantly with increasing age. Among men only the psychic domain decreased significantly with increasing age (see Table 2).

### 3.3. The Impact of Hand Grip Strength and Sociodemographic Factors on Health Related Quality of Life

In a first step the sociodemographic characteristics, contact frequency and hand grip strength were compared according to the total level of health related quality of life. As demonstrated in Table 3 participants with a high health-related quality of life were significantly younger, had significantly more social contacts with non-relatives per month and had the significantly highest grip strength.

Furthermore participants with a high health-related quality of life had a higher level of education, lived exclusively in private homes and were mostly married. They suffered only seldom from chronic diseases and reported no acute disease. Significant associations between sociodemographic factors as well as hand grip strength and the individual domains of health-related quality of could be found. Chronological age correlated significantly negatively with the scores of global, physical, psychic and environmental domain of health-related quality of life. Hand grip strength in contrast correlated significantly positively with the global domain and the physical domain of health-related quality of life. Concerning the number of offspring and intergenerational contact frequency no statistically significant correlations were found, with the exception of a significantly negatively correlation between the number of grandchildren and the physical domain of health-related quality of life (see Table 4). Contact frequency with non-relatives however correlated significantly positively with the global, physical and social domain. These findings were corroborated by the results of the multiple regression analyses (see Table 5). Handgrip strength was significantly positively associated with the scores of the global, physical and environmental domain. Additionally chronological age was negative associated with the physical domain. Furthermore the physical and the social domain were significantly positively associated with the contact frequency with non-relatives. No significant associations were found between health-related quality of life and sex, number of offspring and contact frequency with relatives per month.

## 4. Discussion

Aging is commonly defined as the accumulation of diverse deleterious changes occurring in cells and tissues with advancing age that are responsible for increased risk of disease and death [57]. The recent dramatic increase in the population component of the elderly and the very old makes the preservation of a high health-related quality of life at the population level challenging. Therefore it is is absolutely necessary to identify factors which might preserve physical, psychic and social well-being and enhance a positive ageing process. Several somatic and environmental factors influencing health-related quality of life among elderly have been discussed [12,58,59]. In the present pilot study the impact of physical fitness estimated by grip strength and social contacts with family members and non-relatives have been investigated. Before a detailed discussion of the results can start, some limitations of the present pilot study have to be mentioned. The main shortcoming is the extremely low number of participants. Only 63 men and women participated in this study. We are aware that this low number of participants is the main constraint of this study. This low number is mainly due to the fact that this study was carried out as a pilot study for a larger project concerning health-related quality of life during old age. Consequently all conclusions from the results have to be taken with caution. Another shortcoming is that physical well-being was estimated by hand grip strength only. Nevertheless despite of the low number of participants the significant impact of physical well-being on health-related quality of life could be proved. Hand grip strength was significantly positively related to health-related quality of life. In particular the WHOQOL-BREF global, physical and environmental domains were significantly positively associated grip strength of the dominant hand. This association was true of male as well as female participants. Grip strength decreased significantly with increasing age among men and women. These results are in accordance with those of several other studies. The Hertfordshire cohort study yielded a significant association between higher grip strength and preserved health-related quality of life [24]. The meta-analysis of Rijk et al. [60] showed a high predictive validity of hand grip strength for the decline in cognition, mobility, functional status and mortality among older adults. According to Taekerna et al. [26] poor hand grip strength predicts accelerated dependency, and cognitive decline. Consequently hand grip strength is increasingly seen as an appropriate indicator of physical wellbeing and social, psychic and somatic health [23,25,26,60]. There is no doubt that hand grip strength is first of all a strong indicator of muscle strength and muscle mass. Reduced muscle strength and muscle mass are indicators of the condition of sarcopenia. Skeletal muscle represents the largest component at the tissue-organ level of body composition and it is essential for locomotion, mobility and consequently daily activities. The strong association between skeletal muscle mass and bone mass, which are inter-related throughout life, represents a special problem, and muscle loss and bone loss during old age are inter-related [61,62]. In combination with reduced bone mass i.e., osteoporosis sarcopenia has dramatic consequences such as impaired functional performance, increased risk of falls and, consequently, an increased risk of fragility fractures [8,9,63]. Especially during old age people are vulnerable to the adverse consequences of sarcopenia and osteoporosis, i.e., frailty, increased risk of falls, disability, cognitive impairment depression and even mortality [25,27,55,64]. The decrease of muscle mass and muscle strength is caused by genetic factors [54] but also by lifetime physical activity [55,65], and occupational position [66]. In general the maintenance of muscle strength, physical fitness and physical well-being is an important factor to preserve independence and consequently a high quality of life during old age. The results of the present pilot study corroborate the importance of physical well-being and muscle strength for health-related quality of life during old age.

In contrast to physical well-being indicated by grip strength, the present study found no significant association between intergenerational contacts as well as the number of offspring and health-related quality of life. This finding is in clear contradiction to the results of several previous studies. According to these studies social relations, especially with children and grandchildren, enhance quality of life and subjective well-being during old age [36,37,43,67,68]. Consequently quality of life during old age is seen as a result of social embeddedness in the family [38,39,68]. The family and close intergenerational contacts are not only sources of sociability, family and intergenerational contacts, but also social embeddedness in the family provide a sense of connectedness across generations, linking parents and offspring [69]. Therefore intergenerational contacts have been interpreted as important social variables because close contacts with family members and especially offspring enhance health-related quality of life. These close contacts also enhance health-related quality of live. The present pilot study however could not prove these positive effects of offspring and intergenerational contact frequency on health-related quality of life and is therefore in accordance with studies reporting no association between well-being of the elderly and the contact frequency with their offspring [44,46,47]. This lack of positive association between health- related quality of life and number of offspring as well as intergenerational contacts however may be a result of the extremely small sample size and therefore a statistical artefact. On the other hand family solidarity and close intergenerational contacts are often interpreted as a kind of nostalgia that is no longer true of modern industrialised societies. According to the so called “generation gap” argument elderly and their middle aged children belong to different generations and are therefore quite different in life style, interests and living circumstances [45]. Therefore elderly people may prefer social contacts with friend or other like-minded persons of their own age group. In the present study the contacts with non-relatives had a significantly positive effect on the physical and social domain of health-related quality of life.

## 5. Conclusions

Considering the low number of participants of this pilot study conclusions should be considered with caution, nevertheless a positive effect of physical well-being expressed by hand grip strength and social contacts with non-relatives on health-related quality of life during old age can be assumed.

## Figures and Tables

**Table 1:** Sample description according to sex (χ^2^)/u-test.

	Female	Male	*p*-Value
	x (SD) %	x (SD) %	
Age in years	72.5 (10.1)	72.5 (7.4)	n.s.
**Marital status**			
Single	6.3%	0.0%	n.s.
Married	46.9%	76.2%	
Partnered	6.3%	9.5%	
Divorced	12.4%	9.5%	
Widowed	28.1%	4.8%	
**Living situation**			
Private home alone	40.6%	38.1%	n.s.
Private home together with relatives	46.9%	61.9%	
Home for elderly	12.5%	0.0%	
**Educational level**			
Primary school	9.4%	4.8%	n.s.
Professional training	59.3%	47.6%	
Secondary school	12.5%	19.0%	
College of higher education	9.4%	9.5%	
University degree	9.4%	19.1%	
**Children**	1.6 (0.9)	2.2 (2.5)	0.031
**Grandchildren**	2.3 (1.9)	2.6 (2.5)	n.s.
**Contact frequency with relatives/month**	4.2 (3.1)	3.6 (2.6)	n.s.
**Handgrip strength**			
HGS left	22.67 (7.81)	36.11 (8.73)	0.008
HGS right	23.23 (7.38)	38.33 (7.61)	0.007
**Health related quality of life (domains)**			
global	15.31 (3.82)	15.71(2.12)	n.s.
physical	15.81 (3.57)	16.33 (2.41)	n.s.
psychic	15.69 (2.18)	16.01 (2.41)	n.s.
social	15.08 (2.74)	14.86 (2.64)	n.s.
environmental	17.31 (1.88)	16.81 (2.62)	n.s.

n.s. not significant.

**Table 2:** Hand grip strength and health-related quality of life (domains) according to sex and age (Kruskall-Wallis tests).

n	Female				Male			
	<70 Years	70–79 Years	>80 Years	*p*-Value	<70 Years	70–79 Years	>80 Years	*p*-Value
	16	20	9		7	11	4	
	x (SD)	x (SD)	x (SD)		x (SD)	x (SD)	x (SD)	
Hand grip strength								
HGS dominant hand	26.69 (7.12)	24.14 (4.49)	13.15 (4.33)	<0.001	42.59 (10.11)	36.57 (4.71)	36.19 (7.43)	0.042
Health related quality of life (HRQL)								
global	16.29 (2.58)	16.17 (3.86)	11.33 (4.13)	0.013	16.57 (2.23)	15.82 (1.66)	13.33 (2.31)	n.s.
physical	17.59 (1.68)	16.14 (2.67)	10.95 (4.28)	<0.001	17.88 (1.97)	15.95 (1.95)	14.10 (3.3)	0.048
psychic	16.71 (1.21)	15.06 (1.87)	14.56 (3.51)	0.050	16.95 (2.61)	15.94 (1.80)	14.01 (3.53)	n.s.
social	15.71 (2.35)	14.44 (3.50)	14.39 (1.77)	n.s.	15.67 (3.61)	15.15 (2.09)	14.22 (2.78)	n.s.
environmental	17.93 (1.41)	17.29 (1.64)	15.92 (2.73)	n.s.	17.71 (2.08)	16.55 (2.61)	15.67 (4.07)	n.s.

n.s. not significant.

**Table 3:** Sample characteristics according the level of health-related quality of life. Kruskall-Wallis tests, χ^2^.

	Low HRQL	Medium HRQL	High HRQL	
	Mean (SD)/%	Mean (SD)/%	Mean (SD)/%	*p*-Value
**Sex**				
male	35.3%	38.6%	41.2%	0.939
female	64.7%	61.4%	58.8%	
**Marital status**				
single	0.0%	5.6%	5.9%	0.108
married	41.2%	61.1%	70.6%	
partnered	5.9%	16.7%	0.0%	
divorced	11.7%	5.6%	17.6%	
widowed	41.2%	11.1%	5.9%	
**Living situation**				
Private home alone	41.2%	33.3%	47.1%	0.321
Private home not alone	41.2%	61.1%	52.9%	
Home for elderly	17.6%	5.6%	0.0%	
**Educational level**				
Primary school	17.6%	5.6%	0.0%	0.079
Professional training	70.6%	50.0%	47.1%	
Secondary school	0.0%	22.2%	23.5%	
College	11.8%	11.1%	5.9 %	
University degree	0.0%	11.1%	23.5%	
**Chronic disease**	41.2%	23.5%	12.5%	0.165
**Acute disease**	23.5%	5.9%	0.0%	0.056
**Regular medication**	94.1%	55.6%	64.7%	0.033
**age**	77.8 (8.9)	70.9 (9.5)	69.6 (6.1)	0.012
**Number of children**	1.7 (0.9)	2.0 (1.2)	1.9 (0.9)	0.568
**Number of grandchildren**	3.2 (2.1)	2.4 (2.3)	1.8 (1.8)	0.183
**Offspring contact frequency per month**	2.4 (1.6)	3.1 (3.0)	2.8 (2.3)	0.778
**non-relatives contact frequency per month**	1.8 (0.8)	2.9 (0.9)	3.0 (1.2)	0.001
**HGS dominant hand**	25.1 (10.2)	27.7 (8.8)	33.7 (10.1)	0.037

**Table 4:** Spearman correlations between domains and sociodemographic parameters as well as had grip strength (rho).

	Global	Physical	Psychic	Social	Environmental
Age	−0.41 **	−0.57 **	−0.32 *	−0.09 n.s.	−0.31 *
Number of children	0.10 n.s.	0.02 n.s.	0.06 n.s.	0.16 n.s.	0.01 n.s.
Number of grandchildren	−0.27 n.s.	−0.39 **	−0.17 n.s.	−0.12 n.s.	−0.24 n.s.
Contact frequency relatives	0.22 n.s.	0.23 n.s.	0.23 n.s.	0.04 n.s.	0.24 n.s.
Contact frequency non relatives	0.31 *	0.29 *	0.16 n.s.	0.25 *	0.07 n.s.
HGS dominant hand	0.37 **	0.41 **	0.24 n.s.	0.09 n.s.	0.18 n.s.

* Correlation is significant at the 0.05 level, ** Correlation is significant at the 0.01 level. n.s. not significant.

**Table 5:** Multiple regression analysis for each domain separately: Impact of sex, age, number of offspring, contact frequency and handgrip strength on health related quality of life.

	R^2^	Regression Coefficient B	95% Confidence Interval	*p*-Value
**Domain Global**	0.59			
age		−0.07	−0.18–0.04	0.188
sex		2.15	−0.58–4.88	0.119
Number of offspring		0.49	−0.62–1.59	0.381
Offspring contact frequency per month		0.19	−0.23–0.61	0.371
Contact frequency non relatives		0.79	−0.01–1.59	0.054
HGS dominant hand		0.14	0.00–0.28	0.048
**Domain physical**	0.74			
age		−0.14	−0.23–−0.05	0.003
sex		1.67	−0.48–3.84	0.125
Number of offspring		0.32	−0.56–1.19	0.471
Offspring contact frequency per month		0.01	−0.32–0.34	0.950
Contact frequency non relatives		0.72	0.08–1.36	0.028
HGS dominant hand		0.12	0.01–0.23	0.039
**Domain psychic**				
age	0.43	−0.04	−0.13–0.05	0.364
sex		0.37	−1.79–2.53	0.729
Number of offspring		0.03	−0.85–0.91	0.939
Offspring contact frequency per month		0.02	−0.31–0.35	0.903
non-relatives contact frequency per month		0.56	−0.08–1.19	0.085
HGS dominant hand		0.04	−0.07–0.15	0.495
**Domain social**				
age	0.34	0.03	−0.08–0.15	0.539
sex		0.53	−2.25–3.32	0.701
Number of offspring		0.77	−0.37–1.90	0.180
Offspring contact frequency per month		0.16	−0.26–0.59	0.439
Non-relatives contact frequency per month		0.82	0.01–1.65	0.049
HGS dominant hand		0.04	−0.15–0.14	0.952
**Domain environment**				
age	0.53	−0.04	−0.12–0.05	0.387
sex		1.73	−0.35–3.79	0.100
Number of offspring		0.03	−0.82–0.87	0.946
Offspring contact frequency per month		0.15	−0.17–0.47	0.352
Non-relatives contact frequency per month		0.54	−0.06–1.16	0.077
HGS dominant hand		0.17	−0.04–0.27	0.041
